# Analysis of virus genomes from glacial environments reveals novel virus groups with unusual host interactions

**DOI:** 10.3389/fmicb.2015.00656

**Published:** 2015-07-03

**Authors:** Christopher M. Bellas, Alexandre M. Anesio, Gary Barker

**Affiliations:** ^1^Bristol Glaciology Centre, School of Geographical Sciences, University of BristolBristol, UK; ^2^Cereal Genomics, School of Biological Sciences, University of BristolBristol, UK

**Keywords:** virus ecology, cryosphere, virus genomes, cryoconite, lysogeny, phage plasmid, CRISPR

## Abstract

Microbial communities in glacial ecosystems are diverse, active, and subjected to strong viral pressures and infection rates. In this study we analyse putative virus genomes assembled from three dsDNA viromes from cryoconite hole ecosystems of Svalbard and the Greenland Ice Sheet to assess the potential hosts and functional role viruses play in these habitats. We assembled 208 million reads from the virus-size fraction and developed a procedure to select genuine virus scaffolds from cellular contamination. Our curated virus library contained 546 scaffolds up to 230 Kb in length, 54 of which were circular virus consensus genomes. Analysis of virus marker genes revealed a wide range of viruses had been assembled, including bacteriophages, cyanophages, nucleocytoplasmic large DNA viruses and a virophage, with putative hosts identified as Cyanobacteria, Alphaproteobacteria, Gammaproteobacteria, Actinobacteria, Firmicutes, eukaryotic algae and amoebae. Whole genome comparisons revealed the majority of circular genome scaffolds (CGS) formed 12 novel groups, two of which contained multiple phage members with plasmid-like properties, including a group of phage-plasmids possessing plasmid-like partition genes and toxin-antitoxin addiction modules to ensure their replication and a satellite phage-plasmid group. Surprisingly we also assembled a phage that not only encoded plasmid partition genes, but a clustered regularly interspaced short palindromic repeat (CRISPR)/Cas adaptive bacterial immune system. One of the spacers was an exact match for another phage in our virome, indicating that in a novel use of the system, the lysogen was potentially capable of conferring immunity on its bacterial host against other phage. Together these results suggest that highly novel and diverse groups of viruses are present in glacial environments, some of which utilize very unusual life strategies and genes to control their replication and maintain a long-term relationship with their hosts.

## Introduction

Glaciers and ice sheets host a surprising microbial diversity (Stibal et al., 2006; Hodson et al., 2008; Edwards et al., 2013). Covering 10% of the Earth's land surface, they are arguably a biome in their own right (Anesio and Laybourn-Parry, 2012). Cryoconite holes are water filled depressions found on the surface of the ablation zone of glaciers and ice sheets. The cryoconite material within the holes are hot spots of microbial diversity (Edwards et al., 2013) and activity (Hodson et al., 2008; Telling et al., 2012), despite a constant temperature of ~0.1°C. Diverse viruses are also present and active in these ecosystems (Bellas and Anesio, 2013), with bacteria being subjected to some of the highest rates of virus infection in the literature, where up to 21% of bacterial cells display visual viral infection (Säwström et al., 2007; Bellas et al., 2013). As visible infection only occurs in the final stages, true infection rates are likely to be even higher. Virus-like particles (VLP) are found in abundances of circa 10^8^ VLP g^−1^ cryoconite, and new virus production is approximately 10^7^ VLP g^−1^ h^−1^ (Bellas et al., 2013). Taking burst sizes and bacterial growth rates into account, and assuming lytic interactions, virus production has the potential to lyse bacteria at rates equal to their production or higher. The extreme nature and isolation of these environments, coupled with likely strong selection pressures by viruses (Anesio and Bellas, 2011), make cryoconite viruses an attractive choice to examine virus diversity and functional potential through metagenomics, addressing the hypothesis that novel viruses are present which encoded genomic functions relevant to their environment.

Metagenomics has become an invaluable tool to investigate total viral diversity and functional potential from environmental samples (Angly et al., 2006; Dinsdale et al., 2008; López-Bueno et al., 2009; Hurwitz and Sullivan, 2013). This approach has revealed that the functional potential of viruses has been so remarkable, that in some cases viromes contain similar functional diversity to the corresponding microbial fraction (Dinsdale et al., 2008). There is however, a need to ensure a virome is completely free of non-virus reads before the true functional potential of viruses can be determined (Roux et al., 2013). Further to this, without assembly of virus reads, virus genes cannot be considered together on the same virus. The most popular sequencing choice to generate viral metagenomes has been Roche 454 pyrosequencing technology, as longer read lengths (100–1000 bp over the last decade of virus metagenomics) increased the number of significant matches to databases (Wommack et al., 2008). Recently however, the relatively short (100 bp), but orders of magnitude more reads generated by Illumina technology, coupled with the ability to sequence paired-end reads with variable insert sizes, has allowed for metagenomic assembly of consensus virus genome scaffolds directly from environmental samples (Emerson et al., 2012a; Anantharaman et al., 2014). Whilst these do not represent individual strains from clonal cultures, these scaffolds represent the assembly of a very closely related virus population.

Assembly of virus genomes directly from the environment represents an attractive prospect for several reasons: (1) Analysing virus functional genes in combination with each other, and phage marker genes, allows a detailed analysis of virus lifestyle, function and type; (2) 60–90% of all raw virome reads do not share homology with databases such as GenBank NR (Wommack et al., 2008), assembling phage genome scaffolds therefore represent a way to compare this unknown sequence space between virus genomes; (3) Putative hosts can be assigned to viruses by gene homology and virus attachment sites (Mizuno et al., 2013); (4) The majority of viruses in the environment infect hosts that currently cannot, and may never be cultured. Analysis of genomes from uncultured samples currently represents the only way to unlock their genomes. Thus, assembly represents a way to discovery entirely novel virus groups; (5) Reads from deeply sequenced samples can be mapped to phage genomes from other environments or time points to reveal the presence of phages even if abundance is very low (Emerson et al., 2012b).

In this study we utilized Illumina technology to produce a metagenomic assembly of the virus-size fraction of cryoconite hole ecosystems to examine virus community function and diversity. Unlike the majority of viromes published to date, this study used unamplified DNA. Amplification of DNA before sequencing is often essential to ensure sufficient material for library preparation, however multiple displacement amplification technology is known to preferentially amplify certain sequences (Marine et al., 2014), including significant overrepresentation of ssDNA viruses (Kim and Bae, 2011). Our sequencing library was prepared from unamplified dsDNA filtered through 0.2 μm and therefore reduces bias in the representation of dsDNA virus diversity. This restricted our analysis to this group, which we considered the most important for the first detailed analysis of viruses in this microbially dominated habitat. We utilized a novel bioinformatics approach to select virus scaffolds from cellular contamination, allowing genuine virus functional genes to be analyzed. We created putative annotations of key phages to produce a picture of virus diversity and life strategies in these unique ecosystems.

## Materials and methods

### Sample collection

Samples were collected from the surface of two glaciers in Ny-Ålesund, Svalbard (78°55′ N 11°55′W), Midtre Lovénbreen (ML) and Austre Brøggerbreen (AB) in August 2009, and from the margin of the Greenland Ice Sheet, near Kangerlussuaq (67°9′39.7″N, 50°0′52.7″W) in June 2010. For each sample location, cryoconite was sampled using large sterile syringe into 1 l sterile bags (Whirl-Pak™). Approximately 1 kg of cryoconite was pooled from a 10 m radius for each location and frozen at −20°C for return to the laboratory.

### DNA extraction, sequencing and quality control

Virus DNA extraction is detailed in the Supplementary material. The main stages of the extraction were: (1) Mixing of 700 g sediment with 9 l of phosphate buffered saline (PBS); (2) centrifugation to remove sediment at 2000 × G for 10 min; (3) 0.7 and 0.2 μm filtration of the supernatant; (4) FeCl_3_ precipitation of virus particles (John et al., 2011); (5) concentration via centrifugal concentrators (30 kDa); (6) DNase treatment (50 U, 2 h); (7) Ultracentifugation at 100,000 × G for 20 h through a sucrose cushion; (8) Re-suspension of the pellet in PBS; (9) DNA extraction with a QIAamp MinElute Virus Spin Kit (QIAGEN). The three samples were sequenced in half a lane on an Illumina GAII, with 100 bp paired end reads and an insert size of 400 bp at the Bristol Genomics Facility. Reads were filtered for Illumina adaptors using fastqc-mcf and trimmed to a minimum PHRED score of 20. Both read pairs had to meet the minimum length of 70 bases after quality and adapter trim to be retained.

### Virus diversity (unassembled reads)

An initial metagenomic analysis was conducted on the dataset to determine the virus composition of cryoconite holes. We randomly subsampled 1% of each virome (approximately 600,000 reads) using QIIME (Caporaso et al., 2010) and uploaded the dataset to METAVIR (Roux et al., 2011). Taxonomic comparisons were made by TBLASTX comparisons of reads to NCBI Refseq virus database with an E-value cut-off of 10^−5^. Hits were normalized to the genome length of virotypes via GAAS (Angly et al., 2009).

### Assembly and genome circularity

Each virome was individually imported to CLC Genomics Workbench 6, using a paired read insert size range of 60–650 bp, which was determined from experimental read mapping to test assemblies. Each of the three viromes was assembled five times using k-mer lengths of 24, 33, 43, 53, and 63 and a minimum contig length cut-off of 1000 bp. Reads were mapped back to contigs after the assembly and the contigs were further updated. Contigs over 5000 bp were exported and the five assemblies from each virome concatenated, creating a pooled assembly for each virome. To remove redundancy from the assembly, each pooled assembly was sorted by length before being subjected to a Megablast search of itself using a word size of 100, 97% identity and E-value cut off of 10^−5^. We considered a contig as a match if the blast hit was >1000 bp in length. A custom script was then used to systematically remove smaller contigs which matched larger ones according to this criteria. The list was then used to pick the full fasta entry from the concatenated assembly using QIIME (Caporaso et al., 2010). This allowed for the best k-mer value to be used for each scaffold, achieving the longest assembly. To ensure we were not unnecessarily removing similar viruses from the same assembly, the above redundancy removal parameters were tested on each individual assembly, i.e., 24, 33, 43, 53, and 63 k-mer assemblies separately. In each case, no redundancy was detected with this criteria and no scaffolds were removed. The final scaffolds were uploaded to METAVIR through the assembled contigs pipeline (Roux et al., 2014).

Whilst not all viruses have circular genomes, detecting circularity in a scaffold is considered as a good indication that the genome scaffold is almost complete and that assembly has proceeded correctly. This was done through mapping of paired reads back to scaffolds and screening for discordantly mapped reads. For each virome, paired reads were mapped to all assembled scaffolds simultaneously using bowtie2 short read mapper (Langmead and Salzberg, 2012) using: default sensitive mapping; a paired distance range of 0–900 bp and unaligned reads set to be discarded. The resulting SAM file was filtered for paired reads that discordantly mapped beyond the specified range using SAMtools (Li et al., 2009). Where the discordant mapping was within 1000 bp of the total scaffold length, i.e., the pair mapped to the start and end of the scaffold, it was counted as a circularly mapped pair. If there were three or more circular mapped pairs then the scaffold was counted as circular. Complete circular genomes with overlapping ends were also detected and annotated by METAVIR.

### Database searches and virus confirmation

Scaffolds were subjected to a number of searches to determine if they were viral in origin. Firstly we rejected any scaffold with less than 10 fold coverage to reduce the probability of chimera generation (Luo et al., 2012). Genes were predicted on the scaffolds using GeneMark.hmm with heuristic models (Besemer and Borodovsky, 1999). Predicted amino acid sequences were then compared to seven databases using BLAST or HMMER searches as detailed in Table 1. We initially used hits to the Phage Orthologous Groups (POGs-07-inf.pq) database which contains only genes found in phage to assign some scaffolds as viral (Kristensen et al., 2011). For the remaining scaffolds, further comparison were made to NCBI Refseq virus (ftp://ftp.ncbi.nlm.nih.gov/refseq/release/viral/), Refseq Mitochondrion (ftp://ftp.ncbi.nlm.nih.gov/refseq/release/mitochondrion/), ACLAME's plasmid database (http://aclame.ulb.ac.be/perl/Aclame/Genomes/list.cgi?cat=plasmids). POGs-10 (http://ftp.ncbi.nlm.nih.gov/pub/kristensen/thousandgenomespogs/) and Silva rRNA SSU and LSU (http://www.arb-silva.de/download/arb-files/) as detailed in Table 1. As in all virus metagenomes, the majority of predicted genes have no known predicted function and database matches, therefore when determining the origin of scaffolds, database hits were normalized to the number of HMMER hits to the Pfam-A database. Virus contigs were automatically identified by the following strict parameters: If 50% or more Pfam–A hits also hit to Refseq virus AND 10% or more of the Pfam-A hits also matched POGs10, scaffolds were flagged as viral. Scaffolds were flagged as cellular and manually inspected if one or more hits to Silva SSU/LSU were obtained. Where >50% of Pfam-A hits also hit to ACLAMEs plasmid database, a further BLASTX search against GenBank NR (E-value < 10^−5^) was conducted to manually determine virus identity. Where >50% of Pfam-A hits also hit to Refseq mitochondrion, scaffolds were uploaded and annotated by the MITOS webserver (Bernt et al., 2013).

**Table 1:** **Searches used on all scaffolds to determine if scaffold is viral**.

**Search type**	**Search with**	**Database**	**E-value**	**Criteria for virus**
HMMER	Predicted ORFs	Pfam-A	<10^−5^	–
blastp	Predicted ORFs	Refseq virus	<10^−5^	≥50% of Pfam hits
tblastx	Nucleotide (scaffold)	Refseq mitochondria	<10^−5^	–
blastp	Predicted ORFs	ACLAME plasmids	<10^−5^	<50% of gene hits
blastp	Predicted ORFs	POGs-10	<10^−5^	≥10% of Pfam hits
blastp	Predicted ORFs	POGs-7 infPQ	<10^−5^	≥0
tblastx	Nucleotide (scaffold)	Silva SSU and LSU	<10^−5^	0

### Virome analysis pipelines

Maximum likelihood trees were calculated using the METAVIR pipeline (Roux et al., 2014) for the phage marker genes: TerL (Sullivan et al., 2009); DNA pol B of the Phycodnaviridae (Clasen and Suttle, 2009), DNA pol B (Monier et al., 2008), DNA pol B2 (Drezen et al., 2006), and PhoH (Goldsmith et al., 2011). Bootstrapping was performed using 100 replicates. To annotate functional genes, virus scaffolds were uploaded to MG-RAST (Meyer et al., 2008) using the assembled contigs pipeline and a coverage of one for all scaffolds. Annotation against the subsystems database was performed with an E-value cut-off 10^−5^. To search for clustered regularly interspaced short palindromic repeats (CRISPR). All virus scaffolds were also uploaded to the CRISPRfinder program (http://crispr.u-psud.fr/Server/).

### Whole genome comparison

We compared 54 circular genome scaffolds (CGS) using the methods of Mizuno et al. (2013). We downloaded all tailed phage genomes listed in the International Committee for the taxonomy of viruses (ICTV) database master species list (http://talk.ictvonline.org/files/ictv_documents/m/msl/4911.aspx) from NCBI, plus a selection of dsDNA virus genomes representing other unclassified virus groups (Supplementary Data 2). These genomes were combined with our 54 circular scaffolds and an all vs. all TBLASTX search (BLOSUM45 matrix) was conducted. The resulting bit scores were summed for each genome pair (a successful hit was >30% sequence ID, minimum 30 amino acid length and E-value of <0.01). We computed the Dice coefficient for each genome pair and constructed a dissimilarity matrix according to Mizuno et al. (2013). T-REX was used to construct a neighbor joining tree (Alix et al., 2012) which was drawn in FigTree v1.4.2. (http://tree.bio.ed.ac.uk/software/figtree/).

### Putative host assignment

Lysogenic phages that integrate into the host genomes possess an attachment site (attP) that is an exact match of a host bacterial tRNA gene (attB) (Mizuno et al., 2013). All virus scaffolds were searched (BLASTN) against the tRNADB-CE database (Abe et al., 2014). We considered a hit significant only when it matched with 100% identity on scaffolds that possessed an integrase gene. We used tRNA matches to assign putative host to Phylum or Class level.

## Results

**Figure 1:**
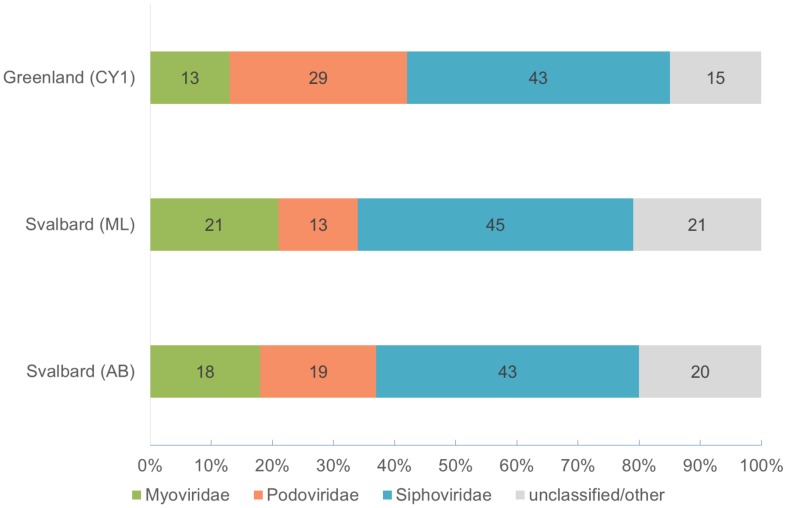
**Virome composition based on BLASTX hits of unassembled reads to the Refseq virus database (E-value < 10^−5^)**. Based on a 0.1% random subsample of each virome. Hits are normalized by genome length of the virotype by GAAS.

Over 21 Gb of sequence data was obtained from the three viromes (Table 2). From the initial analysis of unassembled reads, 0.5–0.8% of all subsampled reads hit to Refseq virus using the METAVIR analysis pipeline. The fact that so few genes had a database match is not unusual for studies of viral metagenomics (Hurwitz and Sullivan, 2013). This is due in part to the limitation of virus databases, the short reads generated for Illumina sequencing and the novelty of the dataset. Of these matching reads, 66–79% hit to the tailed phages (Caudovirales). Amongst the Caudovirales, the Siphoviridae made up the largest group in all three viromes, accounting for 43–45% of matching reads (Figure 1).

**Table 2:** **Illumina read summary and quality control**.

**Reads**	**Greenland (CY1)**	**Svalbard (ML)**	**Svalbard (AB)**
Read pairs	41,205,412	47,705,988	34,093,025
Reads	82,410,824	95,411,976	68,186,050
Passed QC	71,444,796	78,956,224	58,304,468
Gb data	7.22	7.97	5.89

### Optimising assembly

No single k-mer value was suitable for virome assembly, as changing the k-mer length often joined smaller scaffolds together to form circular scaffolds. Therefore, pooling assemblies with multiple k-mer lengths and removing redundancy improved the total amount of sequence data in contigs over 5 kb in each virome (Table S1). After analysis of the data, only scaffolds ≥15 kb were tested for viral origin (or ≥10 kb for circular scaffolds), as smaller scaffolds were too ambiguous to confidently be designated as viruses. Matching this criteria were 3379 scaffolds which were assembled from 8.3 to 26.6% of all reads in the viromes (Table 3). Of these scaffolds, 8.2–30.3% (total 546) were confidently assigned as viruses, these accounted for 8–48% of all reads mapping over 15 kb (1.8–5.6% of all reads in the viromes).

**Table 3:** **Gene prediction and virus scaffold detection from the pooled assemblies**.

	**Greenland CY1 (#)**	**% of genes**	**Svalbard ML (#)**	**% of genes**	**Svalbard AB (#)**	**% of genes**
GeneMark gene predictions	59739		114914		55209	
PfamA gene hits (HMMER scan E-value < 10^−5^)	29228	48.9%	72103	62.7%	31721	57.5%
RefseqVirus gene hits (blastp E-value < 10^−5^)	10520	17.6%	17609	15.3%	8899	16.1%
POGs10 gene hits (blastp E-value < 10^−5^)	4402	7.4%	5632	4.9%	2909	5.3%
POGs7 gene hits (blastp E-value < 10^−5^)	135	0.2%	98	0.1%	82	0.1%
Silva gene hits (tblastx E-value < 10^−5^)	244		385		274	
Scaffolds ≥15 kbp	865		1855		659	
Virus scaffolds ≥15 kb (confirmed)	262	30.3%	152	8.2%	128	19.4%
Reads mapped to all scaffolds ≥15 kb	8,363,805	11.7%	20,979,299	26.6%	4,834,392	8.3%
Reads mapped to virus scaffolds ≥15 kb	4,019,865	5.6%	1677841	2.1%	1077717	1.8%
Reads mapped to other scaffolds ≥15 kb	4,343,940	6.1%	19301458	24.4%	3756675	6.4%
Percentage of reads in scaffolds ≥15 kb which are viral	–	48.1%	–	8.0%	–	22.3%
**CIRCULAR SCAFFOLDS**						
Circular scaffolds ≥10 kb	53		25		16	
Confirmed phage circular scaffolds ≥10 kb	35		12		10	
Circular Mitochondrial scaffolds (MITOS)	5		1		0	
Other cellular origin, plasmid etc.	13		12		6	

### Virus scaffolds

Five-hundred and forty six scaffolds were confirmed as viral in origin, which ranged in size from 10 to 230 kb (Figure S1), representing 684 Mb of virus genome data. Of these scaffolds, 54 were confirmed as circular or circularly permuted, indicating putative consensus genomes had been assembled (Supplementary Data 2). It should be noted however, that not all virus genomes are circular, and many more of our scaffolds are likely complete phage genomes. Our mean virus scaffold length was 30 ± 18 kb (mean ± SD, *n* = 546) (Figure S1) which is in the range of the lower peak of the multi-model distribution of bacteriophage genomes in marine waters of 31–36 kb (Steward et al., 2000). Almost all virus scaffolds appeared novel, predicted genes showed little homology to the Pfam-A database (HMMER E-value < 10^−5^), with 80 ± 11% having no homology to known genes across the three viromes (Supplementary Data 2), (before selection of virus only scaffolds unknown genes made up 37–51% of all predicted genes). Mean coverage of the scaffolds (Figure S1), as determined by read mapping, ranged from 39 to 49 × across the three viromes (noting our minimum cut-off of 10 × coverage for virus assignment). Two scaffolds exhibited exceptional coverage: Circular CY1_33_46 (2500 × coverage; 37,632 bp) accounted for the mapping of 1.1% of all reads from the Greenland (CY1) virome (819,974 of 71,444,796 reads) and 20% of all reads we assign as mapping to viral scaffolds from the same virome; Liner scaffold CY1_53_205 (713 × coverage; 15, 474 bp) mapped 113,372 reads or 0.16% of all reads from Greenland and 2.8% of Greenland reads mapping to viral scaffolds.

All virus scaffolds are publically available and automatically annotated in METAVIR (http://metavir-meb.univ-bpclermont.fr/) under the project name Supraglacial. Circular assembled virus scaffolds are deposited in Genbank under the Bioproject PRJNA283341.

### Non-viral scaffolds

The majority of scaffolds over 15 kb were not viral in origin despite 0.2 μm filtration and purification. Although not the subject of a detailed examination, genomic and mitochondrial DNA was present. Indeed, six circularly mapping scaffolds appeared to be complete mitochondrial genomes with lengths of 42–90 kb, with several more larger contigs showing similarity to mitochondrial genes. Many of the smaller contigs < 15kb contained plasmid replication genes, indicating a significant contribution of plasmid DNA to our viromes.

### Virus functional potential

**Figure 2:**
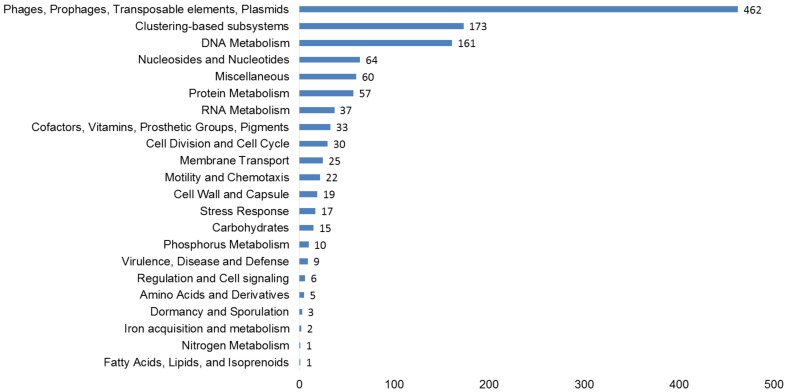
**Functional predictions of all viral genes based on homology to the subsystems database**. The number of hits from our assembled, curated virus database is displayed based on an E-value cut-off <10^−5^ with a minimum of 50% identity.

The functional potential of the three viromes, as determined by homology to the subsystems database was examined on all virus scaffolds. At the subsystems level 1, Phages, Prophages, Transposable elements, and Plasmids made up the largest group, including phage structural, replication, entry, and exit genes (Figure 2). Other typical functional groups for viruses included DNA metabolism (161 hits—DNA replication and repair) and protein metabolism (57 hits), Nucleosides and Nucleotides (64 hits) which includes the common viral auxiliary metabolic gene, ribonucleotide reductase (Sakowski et al., 2014) (37 hits). More unusual, were genes involved in prevents-host-death (Phd) and death-on-curing (Doc) systems (2 hits), phosphate starvation inducible proteins (6 hits), cold shock and heat shock proteins (5 hits), oxidative stress genes (12 hits) and CRISPR associated cas genes (2 hits).

### Virus genome groups

**Figure 3:**
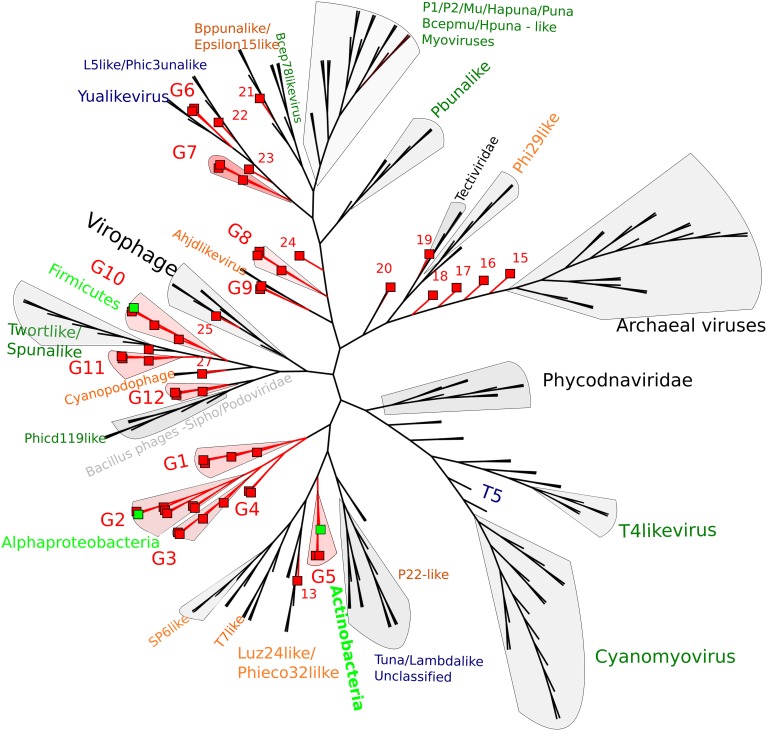
**Whole genome comparison of circular page scaffolds against known phage genomes generated by TBLASTX comparisons**. The tree is constructed with equal branch lengths. Red branches and boxes represent glacial viruses from this study. Red labels represent group number (Supplementary Data 1). Dark green text represent Myoviridae, Orange—Podoviridae, Blue—Siphoviridae. Light green boxes represent a virus scaffold where a putative hosts (light green text) has been assigned by attP homology to the tRNA database.

By using whole genome comparisons between our putative circular genomes and known virus genomes, we grouped 40 CGS into 12 novel virus clusters (Figure 3). A further five CGS grouped with known phage types, and the remaining nine appeared unique. Of the known phage groups (Figure 3): CY1_24_6609 (# 25) grouped with the Virophages; AB_24_7786 (# 13) grouped with the Luz24/Phieco32like Podoviruses; CY1_43_549 (# 21) with Bppunalike and Epsilon15like Podoviruses; ML_53_6570 (# 27) grouped with two Cyanopodophage; CY1_24_7781 (# 20) grouped with the Bacillus phages of the Tectiviridae.

### Putative hosts

**Figure 4:**
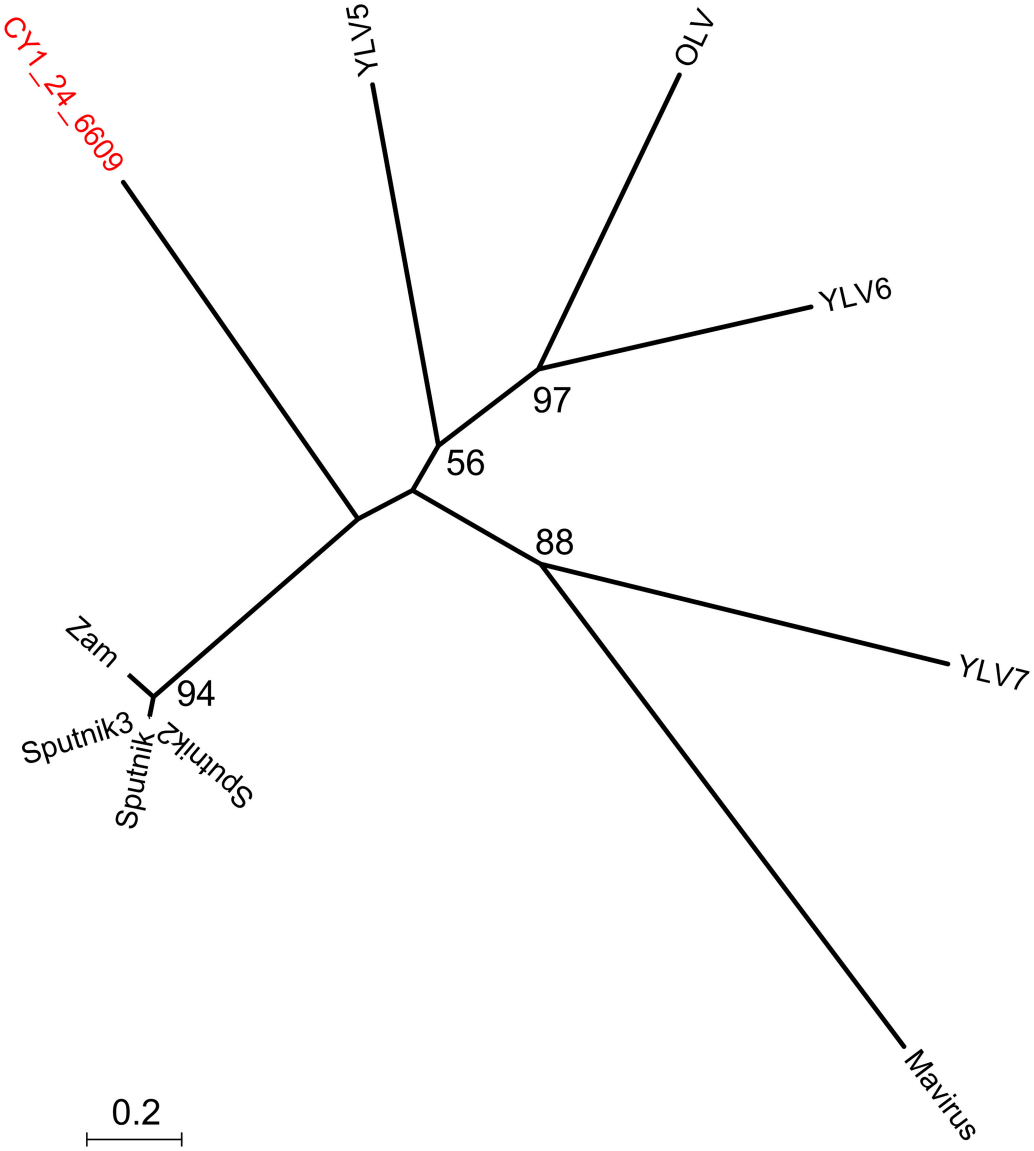
**Phylogeny of the major capsid protein (MCP) gene of the cryoconite virophage**. CY1_24_6609 (#25 Figure 3) is compared with all known virophage. Maximum likelihood tree with 100 bootstrap replicates. Zam, Zamillion virophage; OLV, Organic lake virophage; YLV, Yellowstone lake virophages.

Putative host assignment, determined by matching attP sites from all virus scaffolds to a tRNA gene database, produced 14 exact matches of 28–60 bp (Supplementary Data 2), however we only identified phage integrase genes in seven of these scaffolds and limit our analysis to these (Table 4). Three of these matches were also CGS and are annotated on Figure 3. As tRNA genes are relatively conserved between species, attP site matches were used only for a broad taxonomic assignment. Putative hosts were therefore Actinobacteria, Alphaproteobacteria, Gammaproteobacteria, Firmicutes, and the eukaryotic algal group Haptophyceae (Table 4). Using phage marker genes, another three phages grouped with the cyanophages (Table 4) indicating cyanobacterial hosts (Figures S2, S3). Three scaffolds possessed DNA polymerase family B genes of the Phycodnaviridae (Clasen and Suttle, 2009), further supporting the presence of algal viruses in cryoconite holes (Figure S4). Another three phage possessed major capsid protein marker genes of the NCLDV viruses, implying other eukaryotic hosts may be present. Finally, one scaffold was confirmed as a virophage (Figure 3), of which all known examples in this cluster co-infect with other NCLDV viruses in eukaryotes. Phylogenetic analysis of the V20 major capsid protein gene demonstrated that the cryoconite virophage is unique (Figure 4), however one gene shared homology to the ancient permafrost virus *Pithovirus sibericum* (BLASTX E-value 8 × 10^−11^) which infects amoebae.

**Table 4:** **Putative host assignment for linear and circular virus scaffolds**.

**Phage scaffold**	**Size**		**Putative host**	**Evidence**	**Further information**
CY1_33_1470	32760	Circular	Actinobacteria	attP-attB	Supplementary Date 1
ML_33_9434	17132		Actinobacteria	attP-attB	Supplementary Date 1
ML_53_4264	40814	Circular	Alphaproteobacteria	attP-attB	Supplementary Date 1
CY1_53_17	37329	Circular	Firmicutes	attP-attB	Supplementary Date 1
CY1_43_2205	69796		Gammaproteobacteria	attP-attB	Supplementary Date 1
CY1_43_9289	15483		Gammaproteobacteria	attP-attB	Supplementary Date 1
Ml_53_6570	42514		Cyanobacteria	CGS comparisons	Figure 3 (# 27)
CY1_24_10438	40111		Cyanobacteria	TER_L phylogeny	Figure S2
CY1_24_17307	16195		Cyanobacteria	DNA polB phylogeny	Figure S4
CY1_24_11777	23597		Cyanobacteria	DNA polB phylogeny	Figure S4
AB_33_3099	45797		Algae	DNA pol Phycodnaviridae	Figure S3
AB_43_3071	23682		Algae	DNA pol Phycodnaviridae	Figure S3
ML_43_2588	16549		Algae	DNA pol Phycodnaviridae	Figure S3
ML_24_2669	60149		Algae, Haptophyceae	attP-attB	Supplementary Data 1
AB_33_3238	53511		Eukaryote	MCP of NCLDV	
ML_43_16378	15849		Eukaryote	MCP of NCLDV	
AB_43_3337	15446		Eukaryote	MCP of NCLDV	
Ab_53_508	80133		Eukaryote	24/99 genes match NCLDV	
CY1_24_6609	12595		Virus (NCLDV)	CGS comparisons	Figure 3 (# 25)

*attP-attB, phage and bacterial tRNA attachment sites; MCP, major capsid protein; NCLDV, nucleocytoplasmic large DNA viruses; TER_L, large terminase subunit; CGS, complete genomes acaffold*.

### Plasmid genes

**Figure 5:**
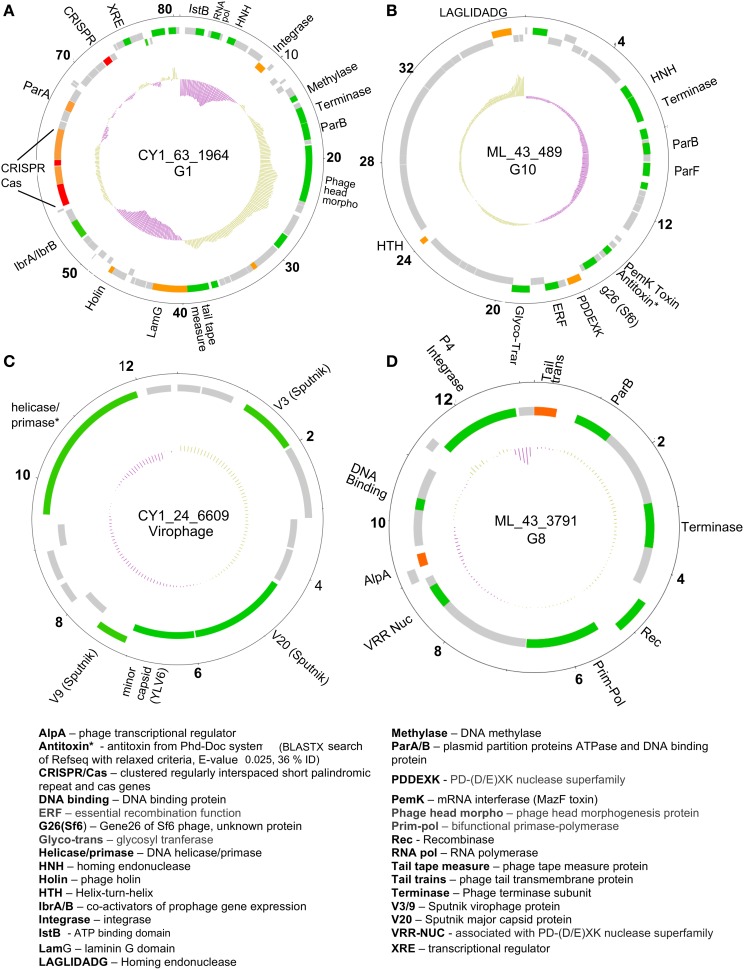
**Representative putative phage genome scaffolds in our assemblies**. Genes were predicted using GeneMark heuristic models and displayed as forward (outer) and reverse (inner) coding. Gray are hypothetical proteins only, green hit to Refseq virus and GenBank NR (BLASTX E-value cut-off 10^−5^), orange hit to GenBank NR only, red are CRISPR arrays. Inner plot shows GC content and numbers denote kilobase pairs. **(A)** Greenland Ice Sheet phage encoding a CRISPR/Cas system and plasmid partition genes. **(B)** Svalbard phage representative of Group 10 (G10), encoding plasmid partition genes and a toxin antitoxin system. **(C)** Greenland virophage with limited homology to Sputnik virophage. **(D)** Group 8 (G8) phage with satellite phage plasmid genome arrangement. Full annotations are given in Supplementary Data 2.

A number of linear and circular scaffolds contained genes that suggested plasmid-like phage replication may occur. These included the plasmid partition genes ParA and/or ParB which ensure low copy number plasmids are distributed to both daughter cells upon cell division. Using HMMER searches against the Pfam databases in METAVIR, 25 phage encoded ParB-like genes, with 13 also encoding the CbiA protein family which includes the ParA family protein. These plasmid-like phage were also represented in our CGS comparison (Figure 3). Group one, eight, and 10 (G1, G8, and G10) contained CGS with multiple plasmid-like genes (Table 5). G1 contained an 80 kb CGS (CY1_63_1964) that encoded both ParA and ParB plasmid partition genes (Figure 5A), as well as a phage integrase, this phage is discussed in the CRISPR phage paragraph below. Group 10 CGS all encoded genes indicative of a lysogenic life strategy, however, 3 of the 4 CGS encoded ParA and/or ParB gene homologs and at least one gene homolog to toxin-antitoxin systems (Table 5; Figure 5B). Such systems, also known as Phd-Doc or plasmid addiction modules ensure any daughter cells that are cured of the phage plasmid are killed (Hazan et al., 2001). Finally, G8 CGS all possessed small (<16 kb) genomes, two of which (CY1_63_1886, ML_43_3791) possessed a primase/polymerase (prim-pol) replication system, similar to satellite phage P4 (Table 5). ML_43_3791 (Figure 5D) also contained a P4 family phage integrase. Similarly, CY1_53_534 contained a phage integrase, phage repressor and primase homologs (Table 5; Supplementary Data 2). The similarities of G8 members to satellite phage P4 replication genes, the small genome size and lack of phage structural genes suggest the G8 members are all satellite phage-plasmids, which rely on other phages for the genes to produce virus particles.

**Table 5:** **Circular genome scaffold (CGS) groups with multiple phage-plasmid-like genes**.

**Virus group and assignment**	**Notable genes (BLASTX of predicted genes, GenBank NR, E-value <10^−5^)**	**Scaffold**	**Length (bp)**	**Coverage**
**G1**				
	VirE (Virulence), DNA pol A	CY1_24_716	37683	225.8
	VirE (Virulence), Terminase, DNA pol A	CY1_24_11561	38498	12.6
Putative phage-plasmid	**ParA, ParB**, Integrase, IbrA/B CRISPR/Cas system	CY1_63_1964	80578	30.2
Lysogenic phage	IbrA, IbrB/**ParBc**, DNA pol A	CY1_24_2481	54228	61.2
**G8**				
Putative satellite phage	Photolyase, Phage Integrase, **Primase**, Cro/Cl	CY1_53_534	14105	18.2
Putative satellite phage	**Prim-pol (P4 like with D5_N region)**, Integrase	CY1_53_1886	15192	22.9
Putative satellite phage	**ParB**, Terminase,Resolvase, Phage tail, **Prim-Pol**, **Integrase(P4)**	ML_43_3791	15770	48.6
**G10**				
Lysogenic phage	Integrase, Terminase	CY1_53_17	37329	92.6
Putative phage-plasmid	**ParA, ParB, Toxin (PemK), Antitoxin**, Terminase	ML_43_489	37582	69.7
Putative phage-plasmid	**Antitoxin (RelB), ParB**, Lipase_GDSL, Terminase	ML_33_2393	38190	40.0
Putative phage-plasmid	**Toxin, Antitoxin (P2 like)**, Integrase, **ParA**, Terminase	CY1_53_144	15551	20.2

*Bold text denotes phage-plasmid-like genes., Cro/Cl, part of phage repressor/antirepressor; VirR, virulence associated protein; IbrA/B, co activators of prophage gene expression; CRISPR, clustered regularly interspaced shore palindromic repeat; P2, phage-plasmid P2; P4, satellite phage P4; ParA/B, Plasmid partition genes; Prim-pol, bifuncational phage-like primase polymerase*.

### Phage encoded CRISPR/Cas system

**Figure 6:**
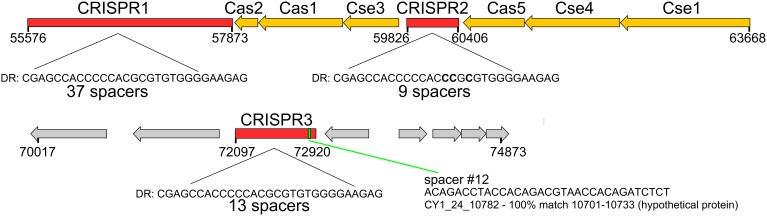
**CRISPR/Cas system found on tailed, lysogenic phage CY1_64_1964**. Cas/Cse are CRISPR associated genes from the type 1-E CRISPR/Cas subtype. Spacer 12 shows the location of the phage matching spacer. DR—Direct Repeat consensus for each CRISPR. Red genes are CRISPR, orange—Cas genes, gray—hypothetical proteins.

Circular genome scaffold CY1_63_1964 of G1 (Figure 5A) possessed three confirmed CRISPR arrays and six cas genes. The direct repeat was 29 bp in length and nearly identical in the three arrays, which contained 37, 9, and 13 spacers, respectively (Figure 6). Cas organization indicated the CRISPR/Cas system belongs to the 1-E (*Escherichia coli*) subtype (Figure 6; Supplementary Data 2). BLASTN searches against all assembled contigs revealed spacer 12 of CRISPR 3 was an exact 32 bp match for phage scaffold CY1_24_10782. The entire CRISPR/Cas system accounted for 11% of the phage genome.

## Discussion

In this study, we have used a novel approach to investigate viromes from natural habitats. By assembling large virus genome fragments and analysing genes together, we have identified multiple hosts and some very unusual ways host and viruses interact at the surface of glaciers and ice sheets. Using metagenomic assembly of the virus-size fraction, we have selected virus scaffolds from cellular contamination, constructing 54 CGS and a further 492 linear scaffolds, many of which could be complete bacteriophage genomes. It should be highlighted however, that each assembled virus genomes scaffold does not represent a clonal isolate, but rather the consensus genome reconstructed from highly similar viruses in the population. In order to be assembled, the virus population must be present in sufficient abundance with sufficient conservation, which suggests large assemblies with high coverage represent actively replicating phage in the environment.

Both whole genome analysis (Figure 3) and virus marker gene phylogeny (Figures S2–S5) indicate that the majority of virus scaffolds assembled in these environments are novel, forming groups independent of the known viruses.

### Non-viral reads

The presence of non-viral DNA in our virus size fraction is worthy of discussion. In this study, some of the largest assembled non-viral contigs were mitochondrial in origin. It is interesting to note that our virus extraction procedure has likely served to concentrate these organelles which presumably originate from abundant Fungi. These mitochondria may have been small enough to pass through the multiple 0.2 μm filter passes, where their outer membranes give them protection from DNase digestion. The remaining genomic and plasmid contamination likely originates from the dissolved fraction, as microscopy checks before DNA extraction showed no cellular contamination. This inability of DNase to digest the dissolved DNA could be due to it being bound to very fine glacial mineral particles, for example it has been previously shown that plasmid DNA is two orders of magnitude more resistant to DNase digestion when bound to minerals (Romanowski et al., 1991).

CsCl gradients may serve to alleviate some of the genomic contamination in future extractions, however CsCl gradients that were tested during the method development in this study failed to concentrate viruses in any one particular gradient interface, presumably due to the large variation in virus types. It is worth noting that whilst CsCl purification techniques are used in many viromes, they are not universally adopted. Our approach to assemble and bioinformatically filter the virome, offers a way to explore virus genomic function in challenging samples such as these, where metagenomic analysis alone would be hindered by cellular contamination (Roux et al., 2013).

### Virus survival strategies

Viruses in polar regions are subjected to challenging conditions for continuous lytic replication, radiation levels are high which can destroy free virions (Suttle and Chen, 1992), burst sizes are low which decreases the probability of new infections (Bellas et al., 2013), and during large parts of the year host production is limited due to snow covering and freeze up of the hole. For these reasons it has been speculated that phage that can switch to a lysogenic life strategy would have a competitive advantage in extreme habitats such as these (Anesio and Bellas, 2011). Lysogenic phage could integrate into their host's genome and replicate silently within their hosts during unfavorable times, switching to lytic replication during short growth bursts in the summer. In our assembled virus scaffolds, although not a quantitative assessment, we identified 49 phage scaffolds containing integrase genes, suggesting this life strategy is important in these systems. Further to this, our metagenomic analysis revealed the largest single group of known phages identified was the Siphoviridae, a phage group that displays the greatest proportion of lysogens from culture experiments (Hambly and Suttle, 2005).

Genome integration is not the only method of lysogeny however, several phages we identified possessed genes indicative of an alternative and unusual strategy, where a phage genome does not integrate into the bacterial chromosome, but exists independently as a plasmid. The ParA/B systems that were identified in 25 of our phage scaffolds are employed by many low copy number plasmids, including the phage P1 plasmid, to ensure that both daughter cells receive a copy of the plasmid upon cell division. Thus, it appears many lysogenic phages in our assemblies may coordinate their replication with cell division, maintaining themselves in a bacterial population without lysis. Three of our CGS in Group 10 (Figure 3) appear to further exhibit both plasmid and phage genes, encoding putative toxin-antitoxin addiction modules (Table 5) and phage-like terminase packaging genes. Toxin-antitoxin systems encode and express a long-lived toxin and a short lived antitoxin in the host which antagonises the negative effects. If the plasmid is not passed on to daughter cells the antitoxin production ceases, leaving the longer lived toxin to cause the death of the bacterium (Hazan et al., 2001). Such proteins are also known as prevents-host-death (Phd) and death-on-curing (Doc) proteins which were also indicated in our functional analysis. In the case of phage P1, the toxin-antitoxin system creates a highly stable long-term relationship with the host (Gazit and Sauer, 1999), where the phage plasmid is only lost in 1 in 10^5^ bacterial generations (Lehnherr and Yarmolinsky, 1995). The majority of predicted proteins in group 10 CGS have no known function, however the genes with known homologs suggest temperate phages with plasmid properties, however the possibility remains that group 10 members are plasmids with phage-like genes.

Further highlighting the uniqueness of our assembled virus genomes, we identified a putative satellite phage group (Figure 3; Table 5) with members sharing gene organization with the satellite phage P4. Phage P4 can propagate via three forms, a lytic tailed virion, an integrated prophage and a plasmid (Briani et al., 2001). P4 does not encoded genes responsible for morphological features, instead it takes advantage of a second, co-infecting lysogenic helper phage P2 to provide structural genes such as tail and capsid genes to allow lytic replication as a virion (Briani et al., 2001). The Group 8 phages appear to follow this structure, possessing mainly replication and integration genes (Figure 5D; Table 5). However, one member, CY1_53_534 is unique in that it also contains a putative DNA photolyase, indicating that this phage may have a role in repairing UV damaged DNA, an ability that may be beneficial in the cryoconite ecosystems which is exposed to high levels of solar radiation.

In summary, the discovery of several unusual phage types and replication systems in cryoconite suggests that multiple life strategies are used by some phages to maintain a stable long-term association with their hosts. Our CGS analysis shows many of these phage fall into two new phage groups, G8 and G10 (Figure 3; Table 5), which highlights the novelty of these phage.

### Phage encoded bacterial immune system (CRISPR/Cas)

A lysogenic phage is often capable of conferring new properties upon its host, such as toxicity (Waldor and Mekalanos, 1996) or enhanced metabolic capability (Lindell et al., 2005; Sullivan et al., 2005). Our CGS dataset contains a lysogenic phage encoding a CRISPR/Cas adaptive bacterial immune system (Figure 6). CRISPRs are found in 40% of all bacterial, and 90% of Archaeal genomes (Horvath and Barrangou, 2010). They consist of a short direct repeat with fixed length spacers, the spacers are usually unique and exactly match regions of phage genomes or other foreign DNA. Where spacers match invading nucleic acid, the region is recognized and cleaved with the aid of CRISPR associated cas genes, providing immunity. New spacers are acquired during phage infection, which provide a record of previous virus infections and can be used to link phages and hosts (Sanguino et al., 2015). However, a handful of phages also possess documented CRISPRs. There is evidence of virus CRISPRs from the human gut virome (Minot et al., 2011), Haloviruses (Garcia-Heredia et al., 2012) and *Clostridium difficile* prophages, where the authors suggest phage encoded CRISPRs could provide superimmunity to their hosts by protecting lysogens against competition from other phage infections (Sebaihia et al., 2006). The best studied example however, comes from *Vibrio Cholera* phage (Seed et al., 2013). In this case, the phage encodes its own CRISPR/Cas system which, in a unique switch, targets and inactivate *Vibrio Cholera's* antiviral genes. In this study, we have identified a lysogenic phage that encodes both an integrase and a plasmid partition ParA-B system, indicating the phage could undergo chromosomal integration or exist as a plasmid (Figure 5A). The phage encodes 3 CRISPRs (Figure 6) with one spacer being an exact match for another phage in our virome CY1_24_10782. To the best of our knowledge this is the first evidence that a phage encoded CRISPR array may target other phage, strengthening the idea that a phage lysogen can confer superimmunity to its bacterial host via CRISPRs. The major difference from classic superimmunity however, is that this protection is not limited to related phages and that immunity can be learned through surviving future phage infections. Our CRISPR containing phage is a member of the tailed bacteriophage (Caudovirales), possessing a large terminase (TerL) subunit (Figure S2), however the spacer matching phage possesses a family B2 DNA polymerase, which places it within a smaller group of known phages. Phylogenetic analysis of the B2 DNA polymerase group of phages places CY1_24_10782 in its own group, out of the Caudovirales (Figure S5), suggesting that the CRISPR phage can provide immunity to its host against other groups of phage. It is also noteworthy that matching spacer is the penultimate spacer in the third repeat-spacer array (Figure 6). CRISPRs normally add new phage spacers to the ends of the CRISPR arrays (Horvath and Barrangou, 2010), suggesting that the matching phage is amongst the most recent phage competition, a fact confirmed by its presence in our assembled virome.

Based on the genes found in our viral scaffold (Figure 5A), we suggest that a lysogenic phage that can be passed onto daughter cells either in the genome, or as a plasmid, which encodes antiviral CRISPRs could be a net benefit to the bacterial host. Negative effects on the host may be outweighed by bringing resistance to lytic phages in the environment, selecting for lysogens and ensuring phage propagation. This strategy may explain why the phage supports a CRISPR/Cas system, using 11% of the phage genome for encoding the CRISPR and associated genes.

### Diverse viruses, diverse hosts

Our analysis of virus genomes from cryoconite has revealed a broad range of microbial hosts. From integrase attachment sites (attP) sites we have identified several bacterial groups, including members of the Alphaproteobacteria, Gammaproteobacteria, Actinobacteria, and Firmicutes, however this analysis was very limited, considering only lysogenic phage. We suspect that the majority of bacterial species are infected by viruses given the high infection frequencies previously observed (Bellas et al., 2013). Phage marker gene analysis further revealed cyanobacteria and algae are also host to viruses in cryoconite. The presence of algal viruses being corroborated by attP-attB homology which indicated at least one host in the Haptophyceae, a group which has previously been detected in cryoconite from the Antarctic dry valleys (Cameron et al., 2012). The unclassified NCLDV viruses in our dataset indicated other eukaryotic hosts are also found, which in turn are also the likely host to a virophage, which we identified in cryoconite holes from the Greenland Ice Sheet (Figure 5C).

Virophages infect the virus production factories of NCLDV viruses during Eukaryotic infection (La Scola et al., 2008). They represent a type of satellite virus, having a negative impact on NCLDV viruses, and even allowing for recovery of their shared hosts in the case of Sputnik virophage, the NCLDV mamavirus and their *Acanthamoeba* host (La Scola et al., 2008). The cryoconite virophage genome scaffold contained gene homologs to Sputnik and Yellowstone Lake Virophage, suggesting an Amoeboid host, therefore its presence points toward a complex interplay between Eukaryotic hosts and their viruses in cryoconite.

To summarize, cryoconite holes possess simple truncated ecosystems, however, the interaction between viruses, their hosts and other competing viruses is complex. Almost all the viruses we have assembled in this study are novel, forming multiple novel virus groups that suggests multiple life strategies and interactions are taking place. These include lysis, lysogeny, plasmid replication with host death-on-curing, satellite phage plasmids which rely on other lysogenic phage for horizontal transmission and even viruses parasitizing on other virus infections. The identification of a phage encoding a bacterial immune system also suggests that some lysogenic phage, in their effort to survive phage competition, may even be beneficial to the bacterial host, providing immunity against other lytic phage infections.

This study has highlighted the benefit of assembling virus genomes from environmental samples to complement virus metagenomics, particularly in novel habitats where the majority of viruses are unknown. By focusing in on the dsDNA viruses, we have demonstrated that this approach is also suitable for challenging samples where cellular DNA contamination is present.

### Conflict of interest statement

The authors declare that the research was conducted in the absence of any commercial or financial relationships that could be construed as a potential conflict of interest.
