# Supplementary material for: Analysis of virus genomes from glacial environments reveals novel virus groups with unusual host interactions
Source: Front Microbiol. 2015 Jul 3;6:656. doi: 10.3389/fmicb.2015.00656 (PMC4490671; doi:10.3389/fmicb.2015.00656)
Supplement: Supplementary file 1 [file DataSheet1.DOCX]

***Supplementary Material***

**Analysis of virus genomes from glacial environments reveals novel virus groups with unusual host interactions**

**Christopher M Bellas^1^*, Alexandre M Anesio^1^, Gary Barker^2^**

^1^Bristol Glaciology Centre, University of Bristol, School of Geographical Sciences, Bristol, UK

^2^Cereal Genomics, University of Bristol, School of Biological Sciences, Bristol, UK

*** Correspondence:** Christopher Bellas, Bristol Glaciology Centre, School of Geographical Sciences, University of Bristol, Bristol, BS8 1SS,UK, email:chris.bellas@bristol.ac.uk

1. **Supplementary Data**

**Table S1 : Virus consensus genome assembly optimisation**

|  | **Greenland (CY1)** | | **Svalbard (ML)** | | **Svalbard (AB)** | |
| --- | --- | --- | --- | --- | --- | --- |
| **Assembly type** | **Count** | **Mb** | **Count** | **Mb** | **Count** | **Mb** |
| 24 k-mer contigs > 5Kb | 3746 | 42.9 | 8055 | 94.1 | 3732 | 38.9 |
| 33 k-mer contigs > 5kb | 3480 | 39.5 | 7665 | 90.3 | 3429 | 35.8 |
| 43 k-mer contigs >5kb | 3125 | 34.8 | 6858 | 80.5 | 2914 | 31.1 |
| 53 k-mer contigs >5kb | 2828 | 29.8 | 5818 | 68.0 | 2270 | 25.2 |
| 63 k-mer contigs >5kb | 2198 | 22.6 | 4705 | 53.6 | 1753 | 17.8 |
| Combined (redundancy removed) | 4345 | 52.1 | 7594 | 104.3 | 4501 | 49.4 |


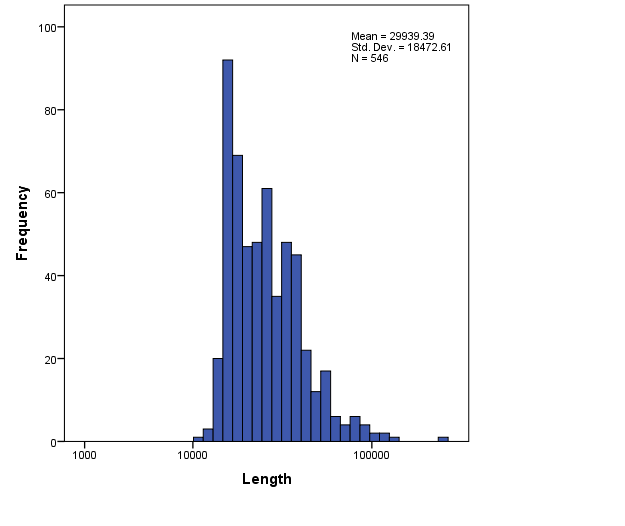

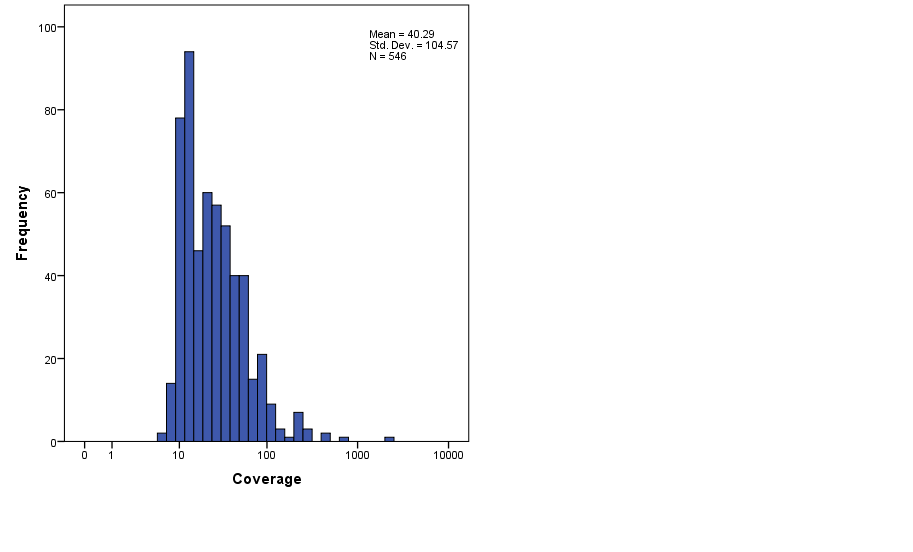


(B)

(A)

**Figure S1**: **Frequency distribution of the length (A) and coverage (B) of virus only scaffolds assembled from all three viromes.**

**Supplementary Materials and Methods**

Virus DNA extraction:

700g of cryoconite sediment was vigorously mixed with 10 litres of 1 x PBS solution in a 20 litre HDPE container (previously soaked in Virkon for 24 hours). The container was mixed for 1 minute every 10 minutes for 1 hour then allowed to settle for 30 minutes. The first 8 litres of supernatant were gently pored off and filtered through multiple 0.7 µm GFF filters (Whatman; previously furnaced at 450°C for 6 hours). For the remaining supernatant with a higher sediment load, multiple rounds of sterile 50 ml centrifuge tubes were filled with supernatant and centrifuged at 2000 x G for 10 mins, before filtration of the supernatant by 0.7 µm GFF filters as before. The 0.7 µm filtrate was then filtered through autoclaved 90 mm, 0.2 µm isopore filters (Whatman).

Viruses were precipitated from the filtrate by FeCl_3_ precipitation following the methods of John et al, 2011. To achieve this, 1 ml of FeCl_3_ solution (0.2 µm filtered) was added to 10 litres of sample and stored at 4 °C overnight. The precipitate was then collected on 4 × 47mm, 0.8 µm polycarbonate filters, which were placed in a 50ml centrifuge tube. We then added 10 ml of pH 7, Oxalate-EDTA buffer (John et al , 2011) with the modification of using Na_2_-EDTA. Filters were stored at 4 °C for 3 days.

Once the precipitate had dissolved, we passed the sample, twice, through a 0.2 µm isopore filter, and loaded onto a Vivaspin (Sartorius) 30 kDa centrifugal concentrator, where it was spun at the maximum recommended speed until the sample volume has been reduced to ~ 1 ml. This was then refilled with 15 ml of PBS and spun down to 1ml, to exchange buffers twice. The sample was then treated with DNase (50 U/ml) for 2 hours then loaded onto 500 µl of 20 % sucrose cushion and ultrafuged for 20 hours at 100,000 × G. The pellet was resuspended in 180 µl of 1 × PBS buffer. To check for cellular contamination, we diluted 1 µl of concentrate with 10 ml PBS and filtered 1ml of the solution onto a 0.02µm Anodisc filter (Whatman). Slides for epifluorescence microscopy were created and stained as detailed in Bellas et al, 2013. DNA was extracted from the concentrate using a QIAamp MinElute virus spin kit (QIAGEN) according to manufactures instructions.

**Supplementary figure captions:**

**Figure S2: Maximum likelihood tree of the virus marker gene TerL.** Large terminase subunit. Generated by METAVIR. Bootstrap values are based on 100 bootstrap replicates. Red labels – this study, the remainder are reference sequences from: Siphoviridae - purple; Podoviridae - orange; Myoviridae - blue; unclassified – green.

**Figure S3: Maximum likelihood tree of the virus marker gene PhoH - Phosphate starvation inducible protein**. Generated by METAVIR. Bootstrap values are based on 100 bootstrap replicates. Red labels – this study, the remainder are reference sequences from: Siphoviridae - dark blue; Podoviridae - pink –; Myoviridae – blue; Phycodnaviridae – orange, unclassified - green.

**Figure S4: Maximum likelihood tree of the virus marker gene AVS.** DNA polymerase of the Phycodnaviridae. Generated by METAVIR. Bootstrap values are based on 100 bootstrap replicates. Red labels – this study, the remainder are reference sequences from: unclassified Phycodnaviridae – light blue; unknown Phycodnaviridae (environmental PCR) – orange; Phaeoviruses – blue; Prasinoviruses – brown; Mimiviridae – pink.

**Figure S5: Maximum likelihood tree of the virus marker gene DNA polB2.** DNA polymerase B2 family. Generated by METAVIR. Bootstrap values are based on 100 bootstrap replicates. Red labels – this study, the remainder are reference sequences from: Salterprovirus – orange; Ampullavirus – green; Tectiviridae – dark blue; Podoviridae – pink; Adenoviridae - light
