# Supplementary figures and images for: Analysis of virus genomes from glacial environments reveals novel virus groups with unusual host interactions
Source: Front Microbiol. 2015 Jul 3;6:656. doi: 10.3389/fmicb.2015.00656 (PMC4490671; doi:10.3389/fmicb.2015.00656)

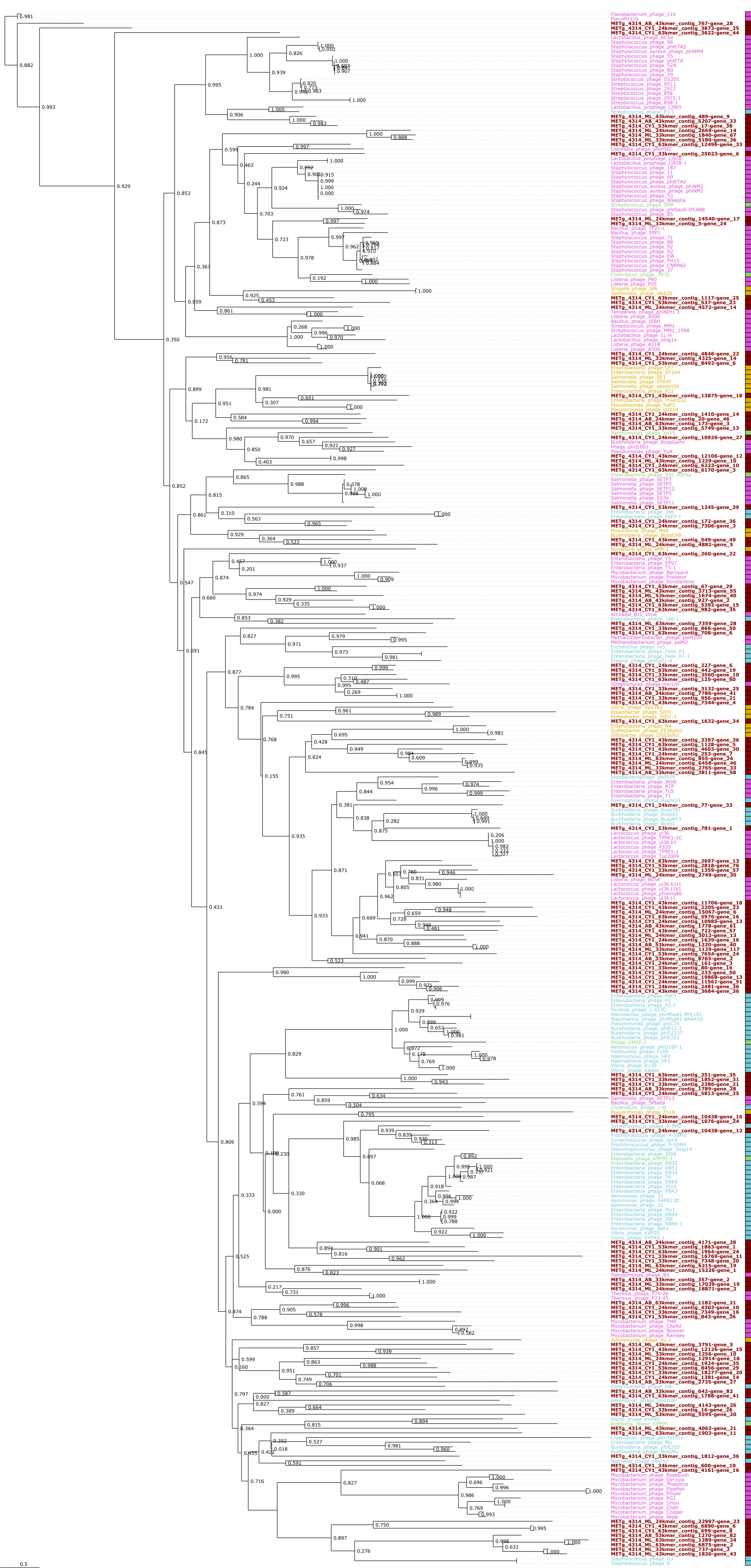

Supplement: Supplementary file 3 [file Image2.PDF]

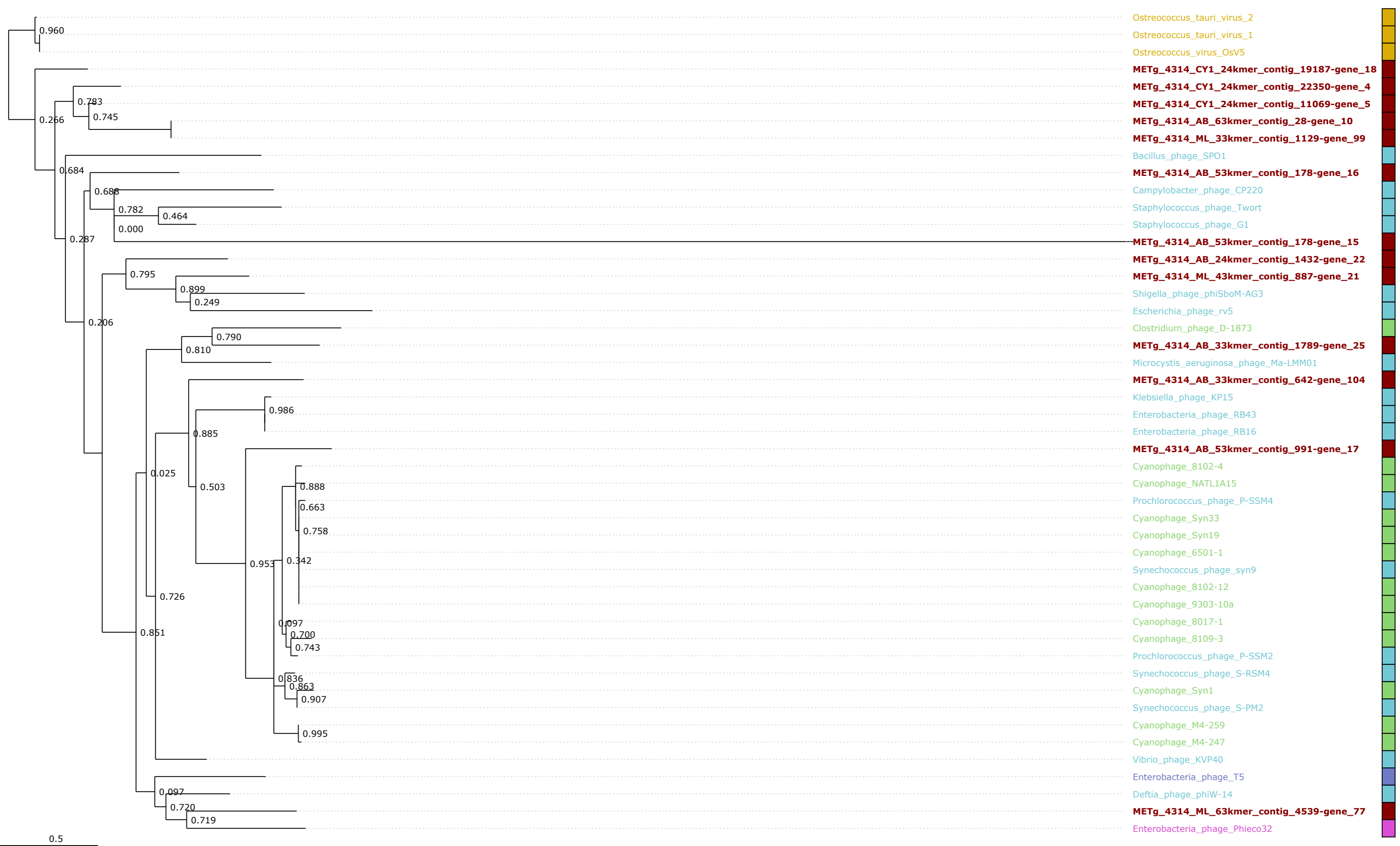

Supplement: Supplementary file 4 [file Image3.PDF]

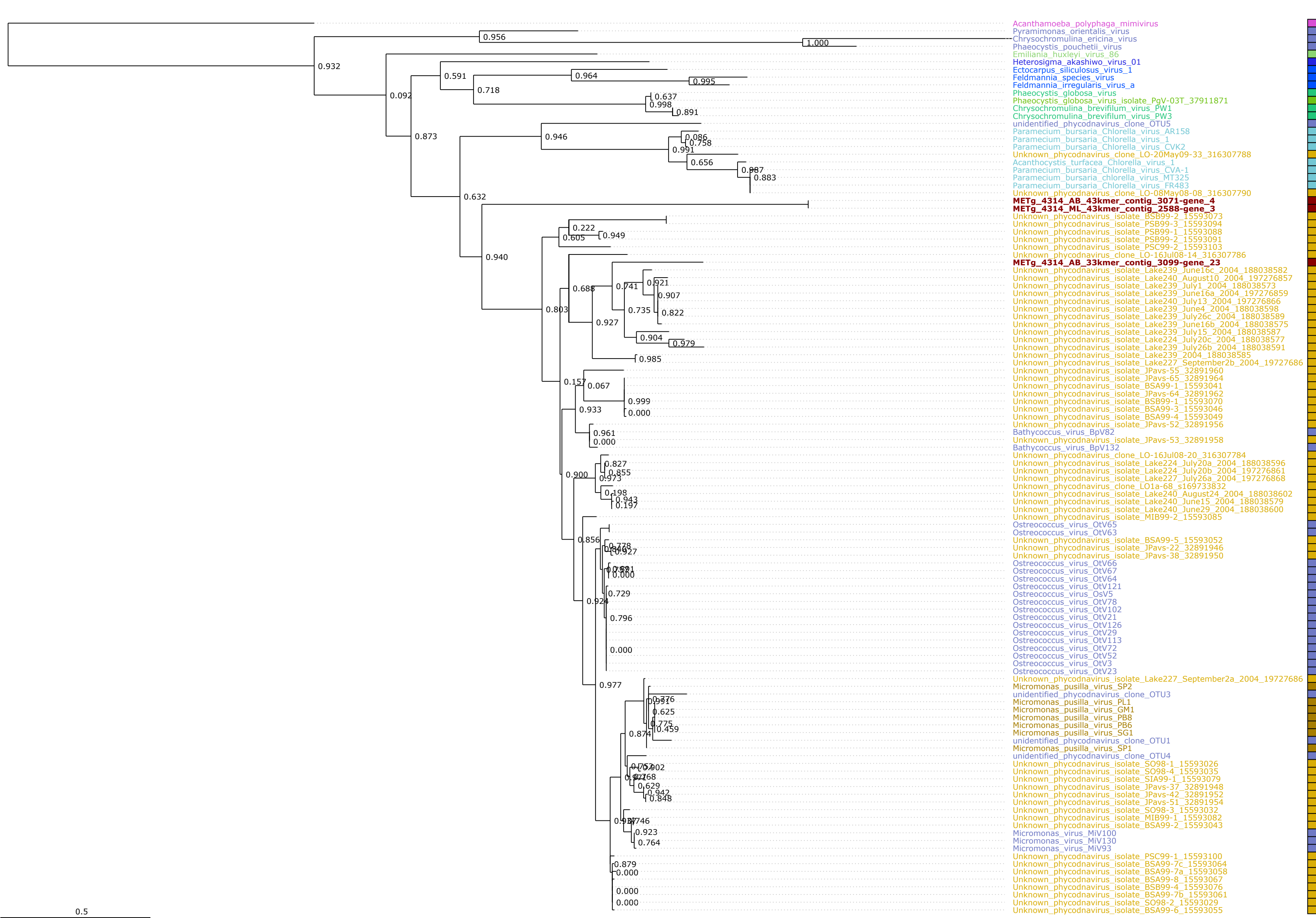

Supplement: Supplementary file 5 [file Image4.PDF]

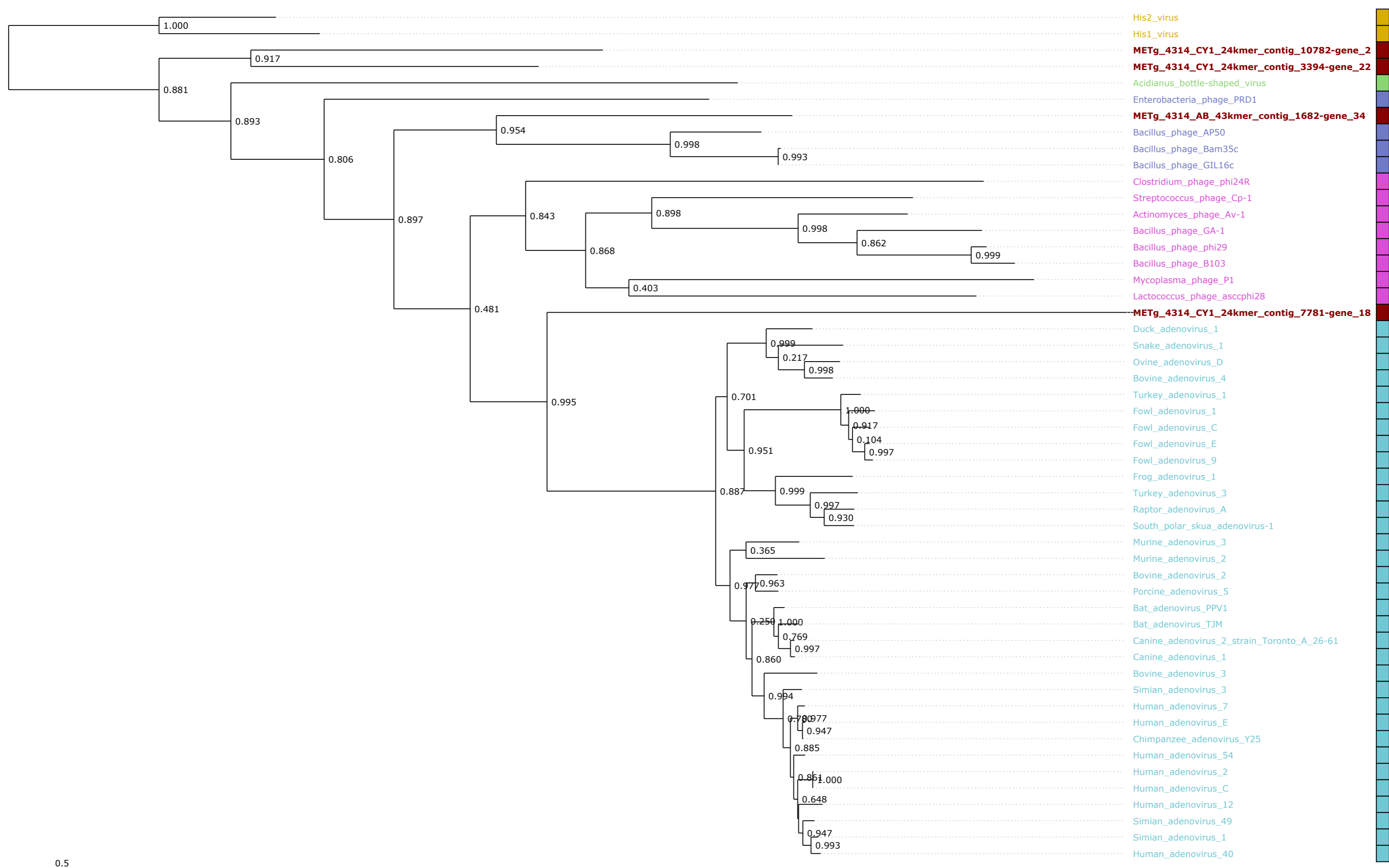

Supplement: Supplementary file 6 [file Image5.PDF]
